# Age-Related Macular Degeneration (AMD) Transmitochondrial Cybrids Protected from Cellular Damage and Death by Human Retinal Progenitor Cells (hRPCs)

**DOI:** 10.1155/2021/6655372

**Published:** 2021-02-09

**Authors:** Jeffrey J. Yu, Daniel B. Azzam, Marilyn Chwa, Kevin Schneider, Jang-Hyeon Cho, Chinhui Hsiang, Henry Klassen, M. Cristina Kenney, Jing Yang

**Affiliations:** ^1^Department of Ophthalmology, Gavin Herbert Eye Institute, University of California Irvine, Irvine, CA 92697, USA; ^2^Department of Pathology and Laboratory Medicine, University of California Irvine, Irvine, CA 92697, USA

## Abstract

**Purpose:**

One of the leading causes of irreversible blindness worldwide, age-related macular degeneration (AMD) is a progressive disorder leading to retinal degeneration. While several treatment options exist for the exudative form of AMD, there are currently no FDA-approved treatments for the more common nonexudative (atrophic) form. Mounting evidence suggests that mitochondrial damage and retinal pigment epithelium (RPE) cell death are linked to the pathogenesis of AMD. Human retinal progenitor cells (hRPCs) have been studied as a potential restorative therapy for degenerative conditions of the retina; however, the effects of hRPC treatment on retinal cell survival in AMD have not been elucidated.

**Methods:**

In this study, we used a cell coculture system consisting of hRPCs and AMD or age-matched normal cybrid cells to characterize the effects of hRPCs in protecting AMD cybrids from cellular and mitochondrial damage and death.

**Results:**

AMD cybrids cocultured with hRPCs showed (1) increased cell viability; (2) decreased gene expression related to apoptosis, autophagy, endoplasmic reticulum (ER) stress, and antioxidant pathways; and (3) downregulation of mitochondrial replication genes compared to AMD cybrids without hRPC treatment. Furthermore, hRPCs cocultured with AMD cybrids showed upregulation of (1) neuronal and glial markers, as well as (2) putative neuroprotective factors, responses not found when hRPCs were cocultured with age-matched normal cybrids.

**Conclusion:**

The current study provides the first evidence that therapeutic benefits may be obtainable using a progenitor cell-based approach for atrophic AMD. Our results suggest that bidirectional interactions exist between hRPCs and AMD cybrids such that hRPCs release trophic factors that protect the cybrids against the cellular and mitochondrial changes involved in AMD pathogenesis while, conversely, AMD cybrids upregulate the release of these neuroprotective factors by hRPCs while promoting hRPC differentiation. These *in vitro* data provide evidence that hRPCs may have therapeutic potential in atrophic AMD.

## 1. Introduction

Age-related macular degeneration (AMD), a progressive retinal condition, ranks as one of the principle causes of irreversible blindness across the world [1, 2]. Epidemiologic studies estimate that 10 million Americans suffer from AMD, comparable to the 12 million with cancer, and surpassing the 5 million with Alzheimer's disease [3–6]. The pathogenesis involves two classifications of AMD, the atrophic (“dry”) form and the exudative (“wet”) form. Dry AMD is a chronic, progressive condition that begins asymptomatically with the extracellular deposition of insoluble drusen aggregates between Bruch's membrane and the retinal pigment epithelium (RPE) [1, 3, 7]. In its advanced stage, this condition then evolves to geographic atrophy, which manifests with degeneration of the RPE and loss of photoreceptors that can cause severe blindness [1, 8]. The less common wet form of AMD emerges and often progresses rapidly in severity. This form is classified by choroidal neovascularization whereby immature blood vessels lead to bleeding and fluid leakage under the retina, causing a sudden loss of central vision [3]. Over 80% of AMD patients are classified as having the dry form, yet these patients may progress to wet AMD, causing more severe loss of vision [9]. Understanding the pathogenesis of AMD is complicated as it involves not only genetic predispositions but also at least four contributing processes, including lipofuscinogenesis, drusogenesis, localized inflammation, and choroidal neovascularization [9].

Therapy for wet AMD includes AREDS formulations and several effective anti-VEGF treatments that are FDA-approved [1, 10, 11]. On the other hand, while eating leafy green vegetables rich in antioxidants is widely recommended for dry AMD, there are no FDA-approved treatments for this condition [1, 12].

The retina is one of the highest oxygen-demanding tissues in the body and relies heavily on the mitochondrial production of ATP via oxidative metabolism [1, 13]. According to the endosymbiotic theory, the mitochondrion is an organelle that evolved from a bacterial ancestor and contains its own genome which is only transferred via the female germline [14, 15]. Mitochondrial DNA (mtDNA) is circular and double-stranded, coding for a variety of key proteins in oxidative phosphorylation [1, 15]. Due to its poor capacity for DNA repair, mtDNA is highly vulnerable to oxidative damage, leading to disruptions in energy metabolism. The result of this is oxidative stress, reduction in antioxidants, and ultimately RPE cell death. Aberrant mitochondrial function and consequent RPE cell death have been linked to a variety of ocular conditions, including AMD, diabetic retinopathy, and glaucoma [16–19].

A variety of cell- and gene-based therapies have emerged as possible restorative treatments for degenerative conditions of the retina that involve the loss of photoreceptors [20]. One reason for the interest in cell-based approaches relates to the poor innate regenerative capacity of the mammalian central nervous system, one consequence of which is that photoreceptor loss is irreversible [21]. Stem cell transplantation has recognized potential not only as a method of retinal cell replacement but also as a means of providing trophic support for host neurons, including photoreceptors [22]. For example, human embryonic stem cell-derived RPE cells have undergone clinical testing in dry AMD by Schwartz et al. [23, 24], while a different cell type, namely, hRPCs, has shown potential in the setting of photoreceptor neuroprotection and associated preservation of visual function in preclinical models of retinal degeneration [25]. In the latter example, the visual benefit is associated with the release of trophic factors from the transplanted hRPCs. Alternatively, RPCs may provide benefits through photoreceptor replacement. Those alternate strategies have led to early stage clinical trials of hRPCs in retinitis pigmentosa (RP) by jCyte (phase 1/2a NCT02320812, phase 2b NCT03073733) and ReNeuron (phase 1/2a NCT02464436).

To our knowledge, there have been no previous studies investigating the role of hRPCs in protecting AMD transmitochondrial ARPE-19 cybrid cells or AMD mitochondria. To better understand the mechanisms by which hRPCs interact with the RPE, we created our transmitochondrial cybrids via fusion of mitochondria-free ARPE-19 *Rho0* cells with platelets, which contain an abundance of mitochondria, isolated from either AMD or age-matched normal patients. Previously, our group has shown that expression levels of RNA and proteins are significantly different in AMD cybrids compared to normal cybrids, despite having identical nuclei in all cell lines [26]. These expression changes are due to differences in mtDNA. Furthermore, our group has shown that AMD cybrids show increased mtDNA fragmentation, impaired levels of expression of mt transcription/replication genes, upregulated proapoptotic genes and proteins, increased mtROS levels, and decreased cellular viability in comparison to normal cybrids [1]. Our most recent studies revealed that the mitochondrial-derived peptide Humanin G and, separately, the antioxidant compound resveratrol protect AMD ARPE-19 cybrids from death [1, 10].

The current study uses a cell coculture system consisting of hRPCs and AMD cybrid cells to test the hypothesis that hRPCs would suppress the expression of harmful genes associated with AMD pathogenesis. We found that the coculture of hRPCs with AMD cybrids resulted in increased cellular viability and decreased expression levels of RNA of the apoptosis, autophagy, endoplasmic reticulum (ER) stress, and antioxidant pathways. Furthermore, coculture resulted in decreased expression of mitochondrial replication and biogenesis genes. Importantly, in examining the effects of AMD cybrids on hRPCs, we found that hRPCs responded to disease AMD cybrids with increased expression of neuroprotective factors and upregulation of glial and neuronal markers. This response was not found when cocultured with age-matched normal cybrids. Our results suggest that a bidirectional interaction occurs between hRPCs and AMD cybrids such that hRPCs release trophic factors that protect the RPE cells against the cellular changes involved in AMD pathogenesis, while AMD cybrids (with their damaged AMD mitochondria) promote the expression of neuroprotective factors by hRPCs as well as differentiation of the multipotent progenitor cells. Together, these *in vitro* data contribute to the mounting evidence that hRPC grafts carry potential as a candidate therapy for atrophic AMD.

## 2. Materials and Methods

### 2.1. Ethics Statement

Research involving human subjects was conducted according to the principles expressed in the Declaration of Helsinki. Informed written consent was obtained, and all research was approved by the Institutional Review Board of the University of California, Irvine (UCI IRB #2003-3131) and UC Irvine Human Stem Cell Research Oversight Committee (UCI hSCRO #2007-5935).

### 2.2. Creation of AMD Transmitochondrial Cybrids

**Figure 1:**
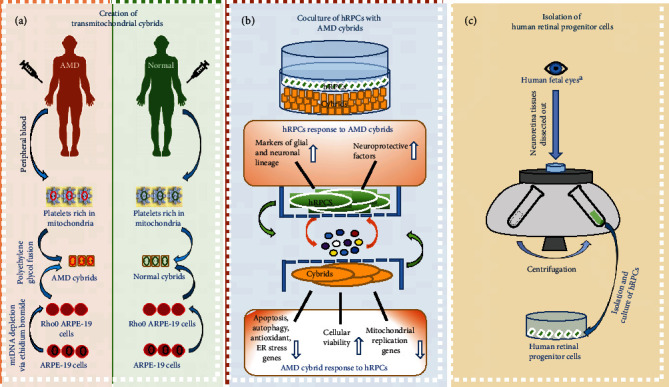
System design. (a) A schematic of the process for creating AMD and normal cybrids using polyethylene glycol fusion of mitochondria-free ARPE-19 (Rho0) cells and mitochondria-rich platelets isolated from peripheral blood of AMD patients or age-matched normal patients. (b) A schematic which illustrates the bidirectional signaling pathway mediated by the coculture of hRPCs with AMD cybrids. (c) A schematic of the process for isolating human retinal progenitor cells from human fetal eyes. ^a^Human fetal eyes (17-20 weeks gestational age) were obtained from the therapeutic termination of pregnancy.

Transmitochondrial cybrids were created as previously described [19]. Polyethylene glycol fusion of mitochondria-free ARPE-19 (Rho0) cells and mitochondria-rich platelets isolated from AMD patients or age-matched normal (AMD, *n* = 5, average age = 80.0 ± 4.5 years; normal, n = 3, average age = 80.7 ± 7.4 years; *p* = 0.88) was performed to create AMD and control cybrids (Figure 1). Epidemiology information for these patients is shown in Table 1. Successful fusion was confirmed with verification of the mtDNA haplogroup profile that compared the original blood sample to the newly created cybrid cell line. Passage 5 transmitochondrial cybrids were used for all experiments.

### 2.3. Isolation of Human Retinal Progenitor Cells

Isolation and culture of hRPCs were performed as previously described [27–29]. Human fetal eyes (17-20 weeks gestational age) were obtained from therapeutic termination of pregnancy. Neuroretina tissues were dissected out and mechanically and enzymatically dissociated by TrypLE (Life Technologies, Grand Island, NY, USA). These cells and cell clusters were then washed, plated, and expanded *in vitro*. At each passage and at isolation, cell number and viability were measured using Trypan blue.

### 2.4. Coculture of Transmitochondrial Cybrids with hRPCs

In each experiment, the AMD cybrids cells were cultured with or without hRPCs. 0.3 million of hRPCs were plated in fibronectin-coated Transwell cell culture inserts with 1 *μ*m pore size (Fisher Scientific, Hampton, NH, USA) and grown in Advanced DMEM/F12 medium supplemented with N2, Glutamax, EGF (20 ng/ml), and bFGF (20 ng/ml) (Life Technologies, Grand Island, NY, USA) at 37°C. Concurrently, 0.5 million of AMD cybrids were plated in six-well plates and grown in DMEM/F12 cell culture media containing 10% FBS at 37°C for 24 hr recovery. After separated incubation for 24 hr, media for both hRPCs and cybrids were replaced with fresh hRPC media. Cell culture inserts containing hRPCs were transferred to the cybrid-containing six-well plates to begin coculture that was incubated at 37°C. After 48 hr coculture, the Transwell inserts containing hRPCs were removed, and hRPCs and cybrids were trypsinized for further experimentation. To study hRPC responses, hRPCs were plated alone or cocultured with cybrids derived from healthy individuals or patients with AMD.

### 2.5. Cell Count and Viability Assay

Following 48 hr culture, samples of trypsinized cocultured and control cybrids were exposed to Trypan blue dye and transferred to slides for cell-counting with the Countess Automated Cell Counter (Invitrogen, Carlsbad, CA, USA). The average numbers of live cells harvested from the treatment and control groups were compared to each other using an unpaired parametric *t*-test.

### 2.6. RNA Extraction and cDNA Synthesis

RNA was extracted from coculture and control cybrids using the RNeasy Mini Kit (QIAGEN, Valencia, CA, USA) following the manufacturer's protocol. Following RNA quantification using NanoDrop 1000 Spectrophotometer (Thermo Scientific, Waltham, MA, USA), cDNA libraries were created by reverse transcription using a Superscript VILO Master Mix (Invitrogen, Carlsbad, CA, USA) or Omniscript RT Kit (QIAGEN Inc., Valencia, CA). cDNA was diluted and stored at -20°C.

### 2.7. Quantitative Reverse Transcription PCR (RT-qPCR)

RT-qPCR was performed using a StepOnePlus Real-Time PCR system and QuantStudio 6 Flex system (Applied Biosystems, Carlsbad, CA, USA). QuantiTect Primer Assays (QIAGEN) and Power SYBR Green PCR Master Mix (Life Technologies, Grand Island, NY, USA) were used.  Table 2 lists the primer information in detail for genes associated with apoptosis (*BAX*, *CASP3*, *CASP7*, and *CASP9*), autophagy (*ATG5*, *ATG12*, *LAMP2*, *LC3B*, and *PARK2*), endoplasmic reticulum stress (*DDIT3* and *XBP1*), antioxidant (*GPX3*, *SOD2*, and *NQO1*), and mitochondrial replication (*POLG*, *POLRMT*, and *TFAM*). A TaqMan assay system was used for hRPC markers: glial lineage (*GFAP*), neuronal lineage (*MAP2*), and neuroprotection (*MDK*, *PTN*, and *FGF2*). Samples were run in triplicate with *HMBS* and *GAPDH*, which were used as a housekeeping gene. The *ΔΔ*Ct method was used to calculate expression fold change between the treatment group and the control group for each cybrid line.

### 2.8. Mitochondrial DNA Copy Number

AMD cybrids (*n* = 5) were plated in six-well plates, and the total DNA was isolated after 48 h coculture using a DNA extraction kit (PUREGENE, QIAGEN, Valencia, CA). In order to determine mtDNA copy numbers with or without hRPC treatment, qPCR was performed using the TaqMan gene expression assays (Cat. # 4369016) with 18S gene to represent nuclear DNA and mt-ND2 gene to represent mtDNA (Cat. # 4331182, Thermo Fisher Scientific). Relative mtDNA copy numbers were determined using the *ΔΔ*Ct method.

### 2.9. Statistical Analysis

Results between treatment and control groups were analyzed for differences by performing one-sample *t*-tests on the expression fold change values from the five cybrid lines for each gene, comparing the values with a hypothetical value of 1 (representing a hypothesis of no difference between the coculture and control groups). Expression fold changes were calculated using fold = 2^−ΔΔCt^. Accordingly, fold values above 1 indicate upregulation of the gene compared to control, while fold values below 1 indicate downregulation of the gene compared to control. Statistical significance was determined at *p* value < 0.05. The fold changes and *p* values for comparison of differential gene expression, mitochondrial copy number, and cellular viability are shown in Table 3.

**Figure 2:**
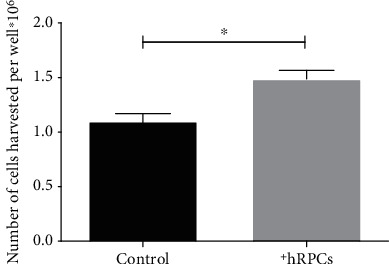
AMD cybrids cocultured with hRPCs show increased cellular viability. Using the Trypan blue dye exclusion assay, the cellular viability of AMD cybrids cocultured with hRPCs at 48 hr increased significantly (*p* = 0.002, *n* = 5) compared to AMD cybrids without hRPCs. Data of average numbers of live cells harvested from the treatment and control groups were compared to each other using an unpaired parametric *t*-test for a sample of *n* = 5.

**Figure 3:**
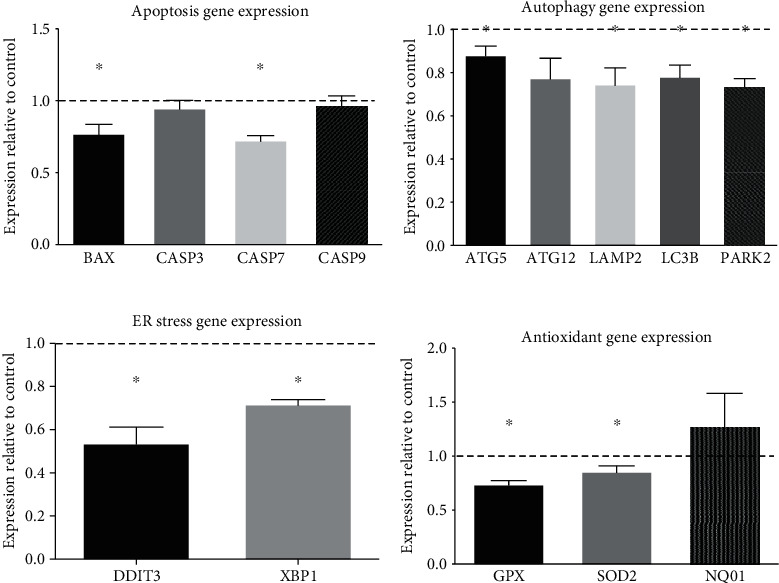
AMD cybrids cocultured with hRPCs show decreased expression levels of apoptosis, autophagy, ER stress, and antioxidant genes at 48 hr. In order to examine the role of hRPCs in protecting AMD cybrids from mtDNA-mediated cellular damage, we used qRT-PCR analyses to measure the differential gene expression profiles of apoptosis, autophagy, ER stress, and antioxidant genes in AMD cybrids with hRPCs versus AMD cybrids without hRPCs. (a–d) In AMD cybrids, hRPCs significantly downregulated *apoptosis genes* (a): *BAX* (77.5% ± 6.3%, *p* = 0.023), *CASP3* (94.9% ± 5.3%, *p* = 0.400), *CASP7* (72.8% ± 3.0%, *p* = 0.001), and *CASP9* (97.4% ± 6.0%, *p* = 0.686); *autophagy genes* (b): *ATG5* (88.3% ± 3.9%, *p* = 0.042), *ATG12* (77.7% ± 9.0%, *p* = 0.068), *LAMP2* (74.8% ± 7.6%, *p* = 0.029), *LC3B* (78.4% ± 5.2%, *p* = 0.014), and *PARK2* (74.0% ± 3.3%, *p* = 0.001); *ER stress genes* (c): *DDIT3* (53.8% ± 7.4%, *p* = 0.003), and *XBP1* (71.9% ± 2.0%, *p* < 0.001); and *antioxidant genes* (d): *GPX* (74.3% ± 3.1%, *p* = 0.001), *SOD2* (86.0%% ± 4.9%, *p* = 0.047), and *NQO1* (128.6% ± 3.0%, *p* = 0.3894) compared to untreated AMD cybrids. Data are represented as mean ± S.E.M., normalized to the control, which is normal cybrids assigned a value of 1 (dotted line). Data between the treatment and control groups were analyzed for differences by performing one-sample *t*-tests on the expression fold change values from the five cybrid lines for each gene (*n* = 5), comparing the values with a hypothetical value of 1.

**Figure 4:**
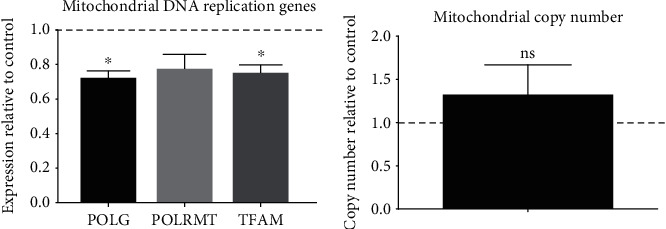
In AMD cybrids, hRPCs decrease the expression of mtDNA replication genes. (a) AMD cybrids cocultured with hRPCs showed significantly reduced expression of mtDNA replication genes. The AMD cybrids with hRPC treatment had lower expression levels of *POLG* (73.0% ± 3.3%, *p* = 0.015) and *TFAM* (72.8% ± 3.0%, *p* = 0.025), but not *POLRMT* (78.9% ± 7.5%, *p* = 0.107) compared to control and untreated AMD cybrids (assigned a value of 1, dotted line). (b) AMD cybrids treated with hRPCs did not show a significant difference in mtDNA copy number compared to control AMD cybrids (copy number relative to control = 1.339 ± 0.329, *p* = 0.361). Data between the treatment and control groups were analyzed for differences by performing one-sample *t*-tests on the expression fold change values either from the five cybrid lines for each gene (*n* = 5) or for mitochondrial DNA copy number (*n* = 5), comparing the values with a hypothetical value of 1.

**Figure 5:**
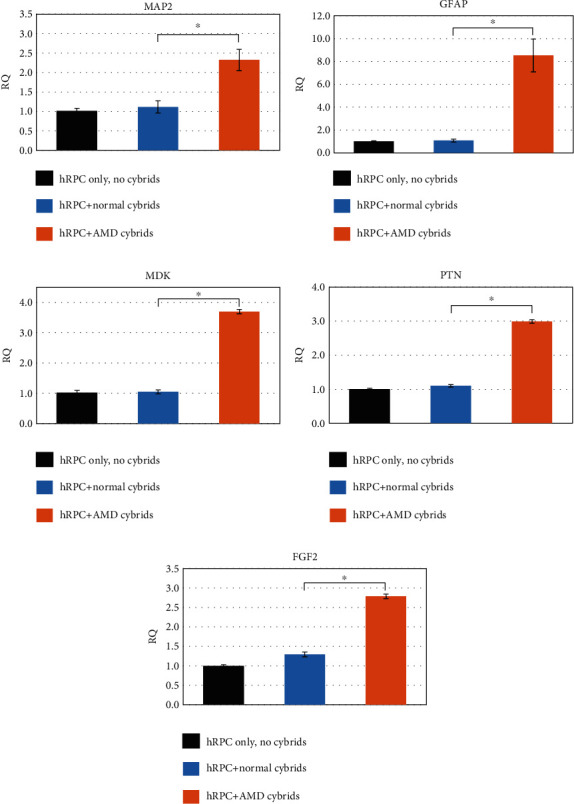
Increased expression of neuronal/glial lineage and neuroprotective genes in hRPCs after coculture with AMD cybrids. In hRPCs, coculture with AMD cybrids significantly increased the expression of (a) markers of neuronal (*MAP2*, fold change = 2.08, *p* < 0.01) and (b) glial lineage (*GFAP*, fold change = 7.93, *p* < 0.01) and neuroprotective factors (c) *MDK* (fold change = 3.53, *p* < 0.01), (d) *PTN* (fold change = 2.71, *p* < 0.01), and (e) *FGF2* (fold change = 2.16, *p* < 0.01) compared to hRPCs with age-matched normal cybrids. The relative quantification (RQ = 2^−ΔΔCt^) was utilized for the data, and Tukey *t*-test and Student *t*-test (unpaired) were used for *p* values between the two groups, with significance determined at *p* < 0.05. See Table 3.

For assessing the hRPC response to diseased AMD cybrids vs. healthy age-matched normal cybrids, the relative quantification (RQ = 2^−ΔΔCt^) was utilized. Tukey *t*-test and Student *t*-test (unpaired) were used for *p* values between two groups, with significance determined at *p* < 0.05. All statistical analyses were performed using Prism, version 7.0 (GraphPad Software Inc.) (Figures 2–4) or JMP (Figure 5).

## 3. Results

### 3.1. Comparison of AMD Cybrids Cocultured with hRPCs (Treatment) versus AMD Cybrids without hRPCs (Control)

#### 3.1.1. AMD Cybrids Cocultured with hRPCs Exhibit Increased Cellular Viability

At 48 h, AMD cybrids cocultured with hRPCs demonstrated a significant increase in viability compared to the control AMD cybrids (Figure 2). The mean number of live cybrid cells harvested per well in the hRPC coculture group was 1.483 × 10^6^ ± 0.082 × 10^6^, while the control AMD cybrids had 1.097 × 10^6^ ± 0.072 × 10^6^ (*p* = 0.0019).

#### 3.1.2. Coculture of AMD Cybrids with hRPCs Decreases Gene Expression of Apoptosis, Autophagy, ER Stress, and Antioxidant Genes in AMD Cybrids

The qRT-PCR was performed to determine the effect of hRPC coculture on the expression of genes involved in cellular damage and death pathways in AMD cybrids. Apoptosis, autophagy, ER stress, and antioxidant genes were downregulated in AMD cybrids cocultured with hRPCs compared to control AMD cybrids grown without hRPC coculture. Two of four apoptosis genes measured were significantly decreased in the treatment group compared to untreated control AMD cybrids (assigned the value of 1 and represented as the dotted line in the figures: *BAX* (77.5% ± 6.3%, *p* = 0.023), *CASP3* (94.9% ± 5.3%, *p* = 0.400), *CASP7* (72.8% ± 3.0%, *p* = 0.001), and *CASP9* (97.4% ± 6.0%, *p* = 0.686)) (Figure 3(a)). Four of five autophagy genes measured showed significantly lower expression levels in the treatment group compared to control: *ATG5* (88.3% ± 3.9%, *p* = 0.042), *ATG12* (77.7% ± 9.0%, *p* = 0.068), *LAMP2* (74.8% ± 7.6%, *p* = 0.029), *LC3B* (78.4% ± 5.2%, *p* = 0.014), and *PARK2* (74.0% ± 3.3%, *p* = 0.001) (Figure 3(b)). Two of two ER stress genes measured were significantly lower in the treatment group compared to the control: *DDIT3* (53.8% ± 7.4%, *p* = 0.003) and *XBP1* (71.9% ± 2.0%, *p* < 0.001) (Figure 3(c)). Two of three antioxidant genes measured were expressed significantly less in the treatment group compared to the control: *GPX* (74.3% ± 3.1%, *p* = 0.001), *SOD2* (86.0% ± 4.9%, *p* = 0.047), and *NQO1* (128.6% ± 3.0%, *p* = 0.3894) (Figure 3(d)).

#### 3.1.3. Effect of hRPCs on AMD Cybrid Mitochondria

Two of three mitochondrial replication genes had significantly lower expression levels in AMD cybrids cocultured with the hRPC cells compared to the control and untreated AMD cybrids (Figure 4(a)): *POLG* (73.0% ± 3.3%, *p* = 0.015), *POLRMT* (78.9% ± 7.5%, *p* = 0.107), and *TFAM* (72.8% ± 3.0%, *p* = 0.025). Mitochondrial DNA copy number (copy number relative to control = 1.339 ± 0.329, *p* = 0.361, Figure 4(b)) was not significantly different between the hRPC treatment and control groups (assigned value of 1, dotted line in graph).

### 3.2. Comparison of hRPCs Cocultured with AMD Cybrids (Treatment) versus hRPCs Cocultured with Normal Cybrids (Control)

#### 3.2.1. hRPC Response to Diseased AMD Cybrids versus Healthy Normal Cybrids

The qRT-PCR was performed to determine the differential gene expression of hRPCs when cocultured with AMD cybrids compared to coculture with age-matched normal cybrids. hRPCs responded to diseased AMD cybrids through upregulation of markers of neuronal (*MAP2*, fold change = 2.08, *p* < 0.01, Figure 5(a)) and glial lineage (*GFAP*, fold change = 7.93, *p* < 0.01, Figure 5(b)). Furthermore, hRPCs responded to the diseased AMD cybrids with elevated expression levels of putative neuroprotective factors: *MDK* (fold change = 3.53, *p* < 0.01, Figure 5(c)), *PTN* (fold change = 2.71, *p* < 0.01, Figure 5(d)), and *FGF2* (fold change = 2.16, *p* < 0.01, Figure 5(e)). On the other hand, hRPCs responded to healthy cybrids with minimal changes that were not statistically significant.

## 4. Discussion

Cell therapy is an emerging therapeutic strategy for various forms of retinal degeneration, most of which are currently untreatable, and in recent years, a number of early stage clinical trials have been initiated [30, 31]. Compared to more conventional approaches, one of the challenges facing cell therapy is to delineate the mechanism of action, which can be complex and difficult to assess using established techniques. This is particularly true in the setting of cytoprotection mediated by innate paracrine effects, such as retinal neuroprotection induced by RPCs. In this study, we use a novel *in vitro* coculture system of hRPCs combined with a transmitochondrial ARPE-19 cybrid model of AMD to investigate the effects of human retinal progenitor cells on gene expression changes and cellular damage seen in AMD. Through cell-based assays and molecular biology techniques, we found that hRPCs suppressed gene expression changes seen in AMD pathogenesis and protected AMD cybrids from cellular damage and death. Additionally, our data showed that hRPCs respond to AMD cybrids through cellular differentiation and increased expression of putative neuroprotective factors. These findings reveal the existence of two-way signaling between hRPCs and AMD cybrids that has potential therapeutic significance, particularly for the use of hRPCs in dry AMD.

Previous studies using mtDNA-deficient *Rho0* ARPE-19 cells have shown that mitochondrial dysfunction plays a role in altering nuclear gene expression related to drusen deposition, inflammation, lipid receptors, and extracellular matrix proteins [1, 32]. This suggests that the oxidative damage to mtDNA in AMD is implicated in disease pathogenesis. The current study utilized the same host cell line of mitochondria-free *Rho0* ARPE-19 cells to create transmitochondrial cybrids containing mitochondria isolated from either AMD patients or age-matched normal patients. Our group previously found that AMD transmitochondrial cybrids showed decreased cellular viability, reduced mtDNA copy numbers, decreased expression of mitochondrial transcription/replication genes, and upregulated gene and protein expression of autophagy, apoptosis, and ER stress compared to cybrids possessing age-matched normal mitochondria [1]. Consequently, these gene expression changes related to RPE cell damage and death in AMD cybrids are attributed to the diseased AMD mitochondria since the nuclei are identical in all of the cybrid cell lines. We previously showed these cybrids to be reliable, personalized models for each patient that are useful for screening mitochondria-targeting drugs [1]. A variety of studies have examined the roles of various treatments as therapeutic targets for AMD, including antithyroid drugs, autophagy regulation, pigment epithelium-derived factor (PEDF), and antioxidant compounds such as esculetin [33–36]. Our most recent studies revealed that the mitochondrial-derived peptide Humanin G and, separately, the antioxidant compound resveratrol protect AMD ARPE-19 cybrids from death [1, 10].

### 4.1. Role of hRPCs in Protecting AMD Cybrids from Cellular Damage and Death

In order to prevent cellular and mitochondrial damage seen in AMD, we hypothesized that coculturing transmitochondrial AMD cybrids with hRPCs would protect the cybrids from cellular damage and death. In that regard, AMD and normal cybrids were cocultured with hRPCs, and the cellular viability was measured. As hypothesized, coculture of AMD cybrids with hRPCs led to a significant increase in numbers of viable cells (*p* = 0.0019), confirming that hRPCs protected AMD cybrids from mitochondria-driven RPE cell death. These results are consistent with previous findings that media from hRPCs inhibited RPE cell death *in vitro*, suggesting that hRPCs secrete antiapoptotic molecules that rescue RPE cells from oxidative damage [37]. Furthermore, Luo et al. found that hRPCs transplanted into the eyes of RCS rats had improved visual acuity and higher cell counts in the outer nuclear layer compared to vehicle-treated control eyes [28].

After confirming the cytoprotective effects of hRPCs on AMD cybrids, we then used qRT-PCR to investigate the role of hRPCs on gene expression changes related to RPE cell death in these cybrids. Gene expression levels of apoptosis, autophagy, ER stress, and antioxidant genes were significantly downregulated in AMD cybrids cocultured with hRPCs compared to control AMD cybrids without hRPC coculture. These findings suggest that coculture with hRPCs prevented the upregulation of genes involved in cellular damage and death pathways in AMD cybrids. Our findings are in agreement with the protective effects of other stem cells on the degenerating retina, such as induced pluripotent stem cell-derived RPE cells [38].

We next examined the effects of hRPC coculture on AMD cybrid mitochondria. AMD cybrids cocultured with hRPCs showed significant downregulation of *POLG* and *TFAM* genes, which are involved in mtDNA replication. Moreover, the mtDNA copy numbers were similar in the hRPC-treated AMD cybrid and untreated AMD cybrids. These findings suggest that the beneficial effects that hRPCs had on the AMD cybrids did not involve increased mtDNA replication and/or mitochondrial biogenesis. It is likely that the hRPCs may modulate the AMD mitochondrion via its other known functions, including changes in oxidative phosphorylation and bioenergetics, or the retrograde signaling (mitochondria to nucleus) that regulates apoptosis along with inflammation pathways and calcium homeostasis. Further studies will be needed to determine the underlying mechanism(s) by which hRPCs rescue the cybrids possessing damaged AMD mitochondria. Other studies have reported reversal of mitochondrial dysfunction in retinal ischemia rats and RPE cells via intravenous mesenchymal stem cells (MSCs) and coculture with MSCs, respectively [39]. Mansergh et al. used retinal progenitor cells as cell therapy to successfully preserve retinal function in Leber's hereditary optic neuropathy, the most prevalent primary mitochondrial disorder [40]. Our findings show that hRPCs are capable of protecting the cybrid cell lines that contain dysfunctional AMD mitochondria, but mechanisms of action are unclear at this time.

### 4.2. Role of AMD Cybrids in hRPC Neuronal/Glial Differentiation and Neuroprotection

Having confirmed the restorative effects of hRPCs on AMD cybrids, we then found that hRPCs responded to AMD cybrids with increased expression of putative neuroprotective factors and upregulation of glial and neuronal markers. Importantly, age-matched normal cybrids were not capable of stimulating retinal progenitor cells in a similar way. Therefore, it appears that the presence of mitochondria from AMD was adequate to recruit hRPCs for protection. Our data suggest that at least some stem-like cells are capable of rescuing RPE cells and that this effect can be induced or amplified by signals from the target cell. In addition to neuroprotection, RPCs might be useful for cell replacement. One application of this approach is suggested by Bartsch et al. from their findings that subretinal transplantation of premature retinal cells not only integrated into the outer nuclear layer but also differentiated into mature photoreceptors [41]. Our results showing hRPC upregulation of glial and neuronal markers indicate that these cells begin to lose multipotency when cocultured with AMD cybrids. While this data does not address possible differentiation into photoreceptors, the induction of differentiation seen could provide benefits in terms of therapeutic safety for a strictly neuroprotective approach by limiting the proliferation of transplanted hRPCs.

In terms of neuroprotection, the basic fibroblast growth factor (bFGF) is a cytokine with known trophic effects in the retina, including rescue of photoreceptors in the RCS rat model [42]. Midkine (MDK) is a cytokine known to play an important role in retinal development [43]. MDK has also been reported to rescue photoreceptors [44] and appears to play a role in modulating the local tissue response to retinal injury [45]. Pleiotrophin (PTN) is a related cytokine that is highly expressed by human neural progenitor cells, including hRPCs [46].

Taken together, these data suggest that a bidirectional interaction exists between hRPCs and AMD cybrids such that hRPCs release trophic factors that are protective against the cellular changes involved in AMD pathogenesis, while AMD cybrids provide signals that result in hRPC differentiation and elevated expression of trophic factors.

### 4.3. New Paradigm for Using a Stem Cell-Based Approach for Atrophic AMD

The mechanism of action by which hRPC coculture exerts a cytoprotective effect on AMD cybrids remains to be elucidated. One possible mechanism may be through protection of elevated numbers of mitochondria. Previous studies demonstrated that AMD mitochondria and primary RPE mitochondria can be rescued by pretreatment with the mitochondrial-derived peptide Humanin [1, 47]. However, our study showed that hRPC coculture did not upregulate mtDNA replication genes or increase mtDNA copy numbers. Another possible mechanism aligns with the early stem cell theory based on stem cell differentiation in the transplantation site, leading to replacement of damaged tissue [40]. Enthusiasm for pursuing this mode of action has tended to wane in recent years in favor of an alternative mechanism involving a paracrine mode of action [48–50]. The premise of the paracrine theory is that stem cell-secreted therapeutic trophic factors provide benefit to injured host tissue via enhancement of natural repair processes, structural repair through physical contact, exertion of a cytoprotective effect (as suggested by decreased gene expression of RPE cell death), and secretion of cytokines, other extracellular proteins, or exosomes. Of note, findings from the current study provide the first evidence that therapeutic-like benefits may be obtained using a stem-cell based approach in atrophic AMD. A combination of hRPC-secreted trophic factors may be aiding recovery from AMD mitochondria-induced RPE damage and therefore may provide a candidate therapy for atrophic AMD. Identification of such paracrine factors would permit testing for therapeutic benefits independent from cell transplantation, which could bypass cell sourcing and a variety of other issues pertaining to cell transplantation.

In conclusion, this study demonstrates the protective role of hRPCs against cell death in AMD transmitochondrial cybrids. Simultaneously, AMD cybrids promote the differentiation of hRPCs and upregulate their expression of putative neuroprotective factors. Our findings support the hypothesis that hRPCs provide a significant cell survival effect with high potential as a candidate therapy for the treatment of atrophic AMD. These results also highlight the bidirectional interaction between hRPCs and AMD cybrids via secretion of specific trophic factors, whose potential beneficial properties should be investigated in future studies. Furthermore, this type of coculture method might have broader use in the setting of assay development, not only for characterization of paracrine interactions and cell therapy product testing, but also for personalized medicine, e.g., to predict individual responses to a particular cell-based drug product. In addition to hRPCs, the method could potentially be adapted to the testing of a range of drug/cell products as well as cellular models of disease indications, not limited to retinal or eye diseases.

## Figures and Tables

**Table 1:** Demographics of the AMD and normal cybrids used in the cellular viability and gene expression experiments.

AMD					Normal				
Cybrid	Haplogroup	Age	Sex	Ethnicity	Cybrid	Haplogroup	Age	Sex	Ethnicity
14.139	H17b	81	F	Caucasian	14.132	H3an	78	M	Caucasian
14.141	H11a2a1	82	F	Caucasian	14.135	H	75	F	Caucasian
14.144	H14a2	86	F	Caucasian	17.193	H	89	M	Caucasian
14.146	H7c6	75	F	Caucasian					
15.155	H5a1	76	M	Caucasian					

Cybrids listed in AMD and normal groups of similar mtDNA haplogroup, age, and ethnicity were used for the experiments. The average ages of AMD compared to normal cybrids are not significantly different, *p* = 0.877.

**Table 2:** Information of the genes related to apoptosis, autophagy, ER stress, and antioxidant genes analyzed in the AMD cybrids. Genes analyzed in the hRPCs represent the neuroprotective factors and markers of glial and neuronal lineage.

Symbol	Gene name	GenBank accession no.	Function
*Apoptosis*			

*BAX*	BCL2-associated X	NM_001291428 NM_001291429 NM_001291430 NM_001291431 NM_004324 NM_138761 NM_138763 NM_138764	This gene encodes a mitochondrially localized protein with conserved B-cell lymphoma 2 homology motifs. Overexpression of the encoded protein induces apoptosis.

*CASP3*	Caspase 3, apoptosis-related cysteine peptidase	NM_004346 NM_032991	Encodes protein as a cysteine-aspartic acid protease that plays a central role in the execution-phase of cell apoptosis.

*CASP7*	Caspase 7, apoptosis-related cysteine peptidase	NM_145248 XM_006725153 XM_006725154 XM_005268295 XM_006725155 XM_005268294 XM_006719962	This gene encodes a member of the cysteine-aspartic acid protease (caspase) family. Sequential activation of caspases plays a central role in the execution-phase of cell apoptosis.

*CASP9*	Caspase 9, apoptosis-related cysteine peptidase	NM_001229 NM_032996	Encodes a member of the cysteine-aspartic acid protease (caspase) family, which is involved in the execution-phase of cell apoptosis.

*Autophagy*			

*ATG5*	Autophagy-related 5	NM_001286106.1 NM_001286107.1 NM_001286108.1 NM_001286111.1 NM_004849.4	The protein encoded by this gene, in combination with autophagy protein 12 functions as an E1-like activating enzyme in a ubiquitin-like conjugating system. The encoded protein is involved in several cellular processes, including autophagic vesicle formation, mitochondrial quality control after oxidative damage, negative regulation of the innate antiviral immune response, lymphocyte development and proliferation, MHC II antigen presentation, adipocyte differentiation, and apoptosis. Several transcript variants encoding different protein isoforms have been found for this gene.

*ATG12*	Autophagy-related 12	NM_001277783.2 NM_004707.4	Autophagy is a process of bulk protein degradation in which cytoplasmic components, including organelles, are enclosed in double-membrane structures called autophagosomes and delivered to lysosomes or vacuoles for degradation. ATG12 is the human homolog of a yeast protein involved in autophagy.

*LAMP2*	Lysosomal-associated membrane protein 2	NM_001122606.1 NM_002294.3 NM_013995.2	The protein encoded by this gene is a member of a family of membrane glycoproteins. This glycoprotein provides selectins with carbohydrate ligands. It may play a role in tumor cell metastasis. It may also function in the protection, maintenance, and adhesion of the lysosome. Alternative splicing of this gene results in multiple transcript variants encoding distinct proteins.

LC3B	Microtubule-associated protein 1 light chain 3 beta	NM_022818.5	The product of this gene is a subunit of neuronal microtubule-associated MAP1A and MAP1B proteins, which are involved in microtubule assembly and important for neurogenesis. Studies on the rat homolog implicate a role for this gene in autophagy, a process that involves the bulk degradation of cytoplasmic component.

PARK2	Parkin RBR E3 ubiquitin protein ligase	NM_004562.3 NM_013987.3 NM_013988.3	The precise function of this gene is unknown; however, the encoded protein is a component of a multiprotein E3 ubiquitin ligase complex that mediates the targeting of substrate proteins for proteasomal degradation. Mutations in this gene are known to cause Parkinson disease and autosomal recessive juvenile Parkinson disease. Alternative splicing of this gene produces multiple transcript variants encoding distinct isoforms. Additional splice variants of this gene have been described but currently lack transcript support.

*ER stress*			

*DDIT3*	DNA damage inducible transcript 3	NM_001195053.1 NM_001195054.1 NM_001195055.1 NM_001195056.1 NM_001195057.1 NM_004083.5	This gene encodes a member of the CCAAT/enhancer-binding protein (C/EBP) family of transcription factors. The protein functions as a dominant-negative inhibitor by forming heterodimers with other C/EBP members, such as C/EBP and LAP (liver activator protein) and preventing their DNA binding activity. The protein is implicated in adipogenesis and erythropoiesis, is activated by endoplasmic reticulum stress, and promotes apoptosis. Fusion of this gene and FUS on chromosome 16 or EWSR1 on chromosome 22 induced by translocation generates chimeric proteins in myxoid liposarcomas or Ewing sarcoma. Multiple alternatively spliced transcript variants encoding two isoforms with different length have been identified.

*XBP1*	X-box binding protein 1	NM_001079539.1 NM_005080.3	This gene encodes a transcription factor that regulates MHC class II genes by binding to a promoter element referred to as an X box. This gene product is a bZIP protein, which was also identified as a cellular transcription factor that binds to an enhancer in the promoter of the T cell leukemia virus type 1 promoter. It may increase expression of viral proteins by acting as the DNA binding partner of a viral transactivator. It has been found that upon accumulation of unfolded proteins in the endoplasmic reticulum (ER), the mRNA of this gene is processed to an active form by an unconventional splicing mechanism that is mediated by the endonuclease inositol-requiring enzyme 1 (IRE1). The resulting loss of 26 nt from the spliced mRNA causes a frame-shift and an isoform XBP1(S), which is the functionally active transcription factor. The isoform encoded by the unspliced mRNA, XBP1(U), is constitutively expressed and thought to function as a negative feedback regulator of XBP1(S), which shuts off transcription of target genes during the recovery phase of ER stress. A pseudogene of XBP1 has been identified and localized to chromosome 5.

*Antioxidant*			

*GPX3*	Glutathione peroxidase 3	NM_002084	The protein encoded by this gene belongs to the glutathione peroxidase family, members of which catalyze the reduction of organic hydroperoxides and hydrogen peroxide (H_2_O_2_) by glutathione, and thereby protects cells against oxidative damage. Several isozymes of this gene family exist in vertebrates, which vary in cellular location and substrate specificity.

*SOD2*	Superoxide dismutase 2	NM_000636	This gene is a member of the iron/manganese superoxide dismutase family. It encodes a mitochondrial protein that forms a homotetramer and binds one manganese ion per subunit. This protein binds to the superoxide byproducts of oxidative phosphorylation and converts them to hydrogen peroxide and diatomic oxygen.

*NQO1*	NAD(P)H quinone dehydrogenase 1	NM_000903.3 NM_001025433.2 NM_001025434.2 NM_001286137.2	This gene is a member of the NAD(P)H dehydrogenase (quinone) family and encodes a cytoplasmic 2-electron reductase. This FAD-binding protein forms homodimers and reduces quinones to hydroquinones. This protein's enzymatic activity prevents the one electron reduction of quinones that results in the production of radical species. Mutations in this gene have been associated with tardive dyskinesia (TD), an increased risk of hematotoxicity after exposure to benzene, and susceptibility to various forms of cancer. Altered expression of this protein has been seen in many tumors and is also associated with Alzheimer's disease (AD). Alternate transcriptional splice variants, encoding different isoforms, have been characterized.

*Mitochondrial replication*			

*POLG*	DNA polymerase gamma, catalytic subunit	NM_001126131.2	This gene encodes the catalytic subunit of mitochondrial DNA polymerase. Defects in this gene alter the replication of mitochondrial DNA and may cause progressive external ophthalmoplegia with mitochondrial DNA deletions 1 (PEOA1), among other diseases.

*POLRMT*	RNA polymerase mitochondrial	NM_005035.4	The polypeptide encoded by this gene is a mitochondrial DNA-directed RNA polymerase. The role of the gene product is mitochondrial gene expression and providing RNA primers to initiate replication of the mitochondrial genome.

*TFAM*	Transcription factor A, mitochondrial	NM_001270782.1	This gene encodes an important mitochondrial transcription factor. This encoded protein also plays a role in mitochondrial DNA replication and repair. Alzheimer's and Parkinson's diseases have been associated with sequence polymorphisms in this gene.

*Glial marker*			

*GFAP*	Glial fibrillary acidic protein	NM_001131019.3	The protein encoded by this gene is a major intermediate filament protein of mature astrocytes. This protein is utilized as a marker during development to distinguish astrocytes from the other glial cells. Gene mutations cause a disorder called Alexander disease, which affects astrocytes in the central nervous system. The result of alternative splicing is distinct isoforms encoded by multiple transcript variants.

*Neuronal marker*			

*MAP2*	Microtubule-associated protein 2	NM_001039538.2	The protein encoded by this gene belongs to the family of microtubule-associated proteins. Proteins in this family are involved in microtubule assembly, an essential step in neurogenesis. Similar genes in rat and mouse encode neuron-specific cytoskeletal proteins in dendrites, suggesting a role in determining dendritic shape during the development of the neuron. Several distinct isoforms encoded by alternatively spliced variants have also been described.

*Neuroprotective factors*			

*MDK*	Midkine	NM_001012333.2	The protein encoded by this gene is part of the small family of secreted growth factors that has the ability to bind heparin and respond to retinoic acid. This encoded protein functions to promote cell growth, migration, and angiogenesis, which is most prominent during tumorigenesis. This gene has been studied as a therapeutic target in a variety of disorders. Multiple isoforms encoded by alternatively spliced transcript variants have been described.

*PTN*	Pleiotrophin	NM_001321386.2	This gene encodes a secreted heparin-binding growth factor. The encoded protein functions in cell growth and survival and migration, as well as angiogenesis and tumorigenesis. Multiple transcript variants result from alternative splicing and alternative promoters.

*FGF2*	Fibroblast growth factor 2	NM_001361665.2	This gene encodes a protein that is part of the family of fibroblast growth factors (FGF). Members of the FGF family bind heparin and function in broad mitogenic and angiogenic activities. The gene product has been implicated in a diverse array of biological processes, including development of the limbs and nervous system, tumor growth, and wound healing. The mRNA of this gene has multiple polyadenylation sites and results in five different isoforms due to alternative translation from non-AUG (CUG) and AUG initiation codons. The isoforms initiated by CUG are localized in the nucleus and play a role in the intracrine effect, whereas the form initiated by AUG is typically in the cytosol and plays a role in this FGF's paracrine and autocrine effects.

*Housekeeping gene*			

*HMBS*	Hydroxymethylbilane synthase	NM_000190.4	The protein encoded by this gene is part of the hydroxymethylbilane synthase superfamily. The gene product is the third enzyme in the heme biosynthetic pathway and serves as a catalyst for the head to tail condensation of four molecules of porphobilinogen into the linear hydroxymethylbilane. Gene mutations are linked to the autosomal dominant disease acute intermittent porphyria.

*GAPDH*	Glyceraldehyde-3-phosphate dehydrogenase	NM_001256799.3	The protein encoded by this gene is part of the glyceraldehyde-3-phosphate dehydrogenase family. The gene product catalyzes an important step in the metabolism of carbohydrates, the oxidative phosphorylation of glyceraldehyde-3-phosphate when in the presence of both nicotinamide adenine dinucleotide (NAD) and inorganic phosphate.

*MT-ND2*	Mitochondrially encoded NADH dehydrogenase 2	NC_012920.1	The gene product is a core subunit of the NADH dehydrogenase complex in the mitochondrial membrane respiratory chain. This complex plays a role in the electron transfer from NADH to the respiratory chain. Ubiquinone is believed to be the immediate electron acceptor for this enzyme. Diseases in association with this gene include Leber optic atrophy.

*RNA18SN5*	RNA, 18S ribosomal N5	NR_003286.4	The 45S ribosomal DNA (rDNA) clusters are designated as RNR1 through RNR5 on chromosomes 13, 14, 15, 21, and 22, respectively. The 445S rDNA has a repeat unit encoding a 45S rRNA precursor that gets processed to produce the 18S, 5.8S, and 28S rRNAs. As such, this gene is a representative copy of 18S rRNA, whose chromosomal location is not known.

**Table 3:** qRT-PCR analysis for differential gene expression, mitochondrial copy number, and cellular viability for AMD/hRPC vs. AMD and hRPC/AMD vs. hRPC/normal cybrids.

AMD cybrids+hRPCs (Tx) vs. AMD cybrids (CTRL)				hRPCs+AMD cybrids (Tx) vs. hRPC+normal cybrids (CTRL)						
Gene	Function	Tx vs. CTRL fold	Tx vs. CTRL *p* value	Gene	Function	hRPC only	hRPC+normal cybrids	hRPC+AMD cybrids	Tx vs. CTRL fold	Tx vs. CTRL *p* value
*BAX*	Apoptosis	0.78	0.023	*MAP2*	Neuro. marker	1.02 ± 0.07	1.12 ± 0.16	2.32 ± 0.27	2.08	0.0015
*CASP3*	Apoptosis	0.95	0.400	*GFAP*	Glial marker	1.01 ± 0.06	1.08 ± 0.13	8.55 ± 1.44	7.93	<0.0001
*CASP7*	Apoptosis	0.73	0.0001	*MDK*	Neuroprotect.	1.02 ± 0.07	1.05 ± 0.07	3.69 ± 0.07	3.53	<0.0001
*CASP9*	Apoptosis	0.97	0.686	*PTN*	Neuroprotect.	1.00 ± 0.02	1.10 ± 0.04	2.99 ± 0.06	2.71	<0.0001
*ATG5*	Autophagy	0.88	0.042	*FGF2*	Neuroprotect.	1.00 ± 0.02	1.29 ± 0.06	2.78 ± 0.06	2.16	<0.0001
*ATG12*	Autophagy	0.78	0.068							
*LAMP2*	Autophagy	0.75	0.029							
*LC3B*	Autophagy	0.78	0.014							
*PARK2*	Autophagy	0.74	0.001							
*DDIT3*	ER stress	0.54	0.003							
*XBP1*	ER stress	0.72	0.0001							
*GPX*	Antioxidant	0.74	0.001							
*SOD2*	Antioxidant	0.86	0.047							
*NQO1*	Antioxidant	1.29	0.389							
*POLG*	Mito. repl.	0.73	0.015							
*POLRMT*	Mito. repl.	0.79	0.107							
*TFAM*	Mito. repl.	0.73	0.025							
	Mito. copy #	1.34	0.361							
	Cell viability	1.27	0.0019							

*N* = 5 with 10 values for each sample. Fold = 2^−ΔΔCT^. Fold values above 1 indicate upregulation of the gene compared to control. Fold values below 1 indicate downregulation of the gene compared to control. Controls are assigned a value of 1. Comparisons were done using one-sample *t*-tests. AMD: age-related macular degeneration; hRPC: human retinal progenitor cells; Tx: treatment; CTRL: control.

## Data Availability

The data used to support the findings of this study are available from the corresponding author upon reasonable request.
